# Pathogenesis of natural and experimental *Pseudorabies virus* infections in dogs

**DOI:** 10.1186/s12985-015-0274-8

**Published:** 2015-03-18

**Authors:** Letian Zhang, Cheng Zhong, Jushi Wang, Zijie Lu, Lei Liu, Wanlian Yang, Yanli Lyu

**Affiliations:** College of Veterinary Medicine, China Agricultural University, 100193 Beijing, P R China; China Animal Husbandry Group, 100070 Beijing, P R China

**Keywords:** Canine, Pseudorabies virus, Pathogenesis, Cardiac injury, Hemorrhage, Ganglioneuritis

## Abstract

**Background:**

Since late 2011, cases of suspected canine pseudorabies have increased in north China with the outbreak of swine pseudorabies in the same area, but the pathogenesis of canine *Pseudorabies virus* (PRV) infections in China is poorly understood. In this study, we investigated the pathogenesis of canine pseudorabies.

**Methods:**

The pathological changes in 13 dogs that died of natural PRV infections (confirmed by pathogen detection) during 2011–2013 in Beijing were evaluated. An experimental study was also conducted in which healthy adult beagle dogs were administered PRV isolate BJ-YT by subcutaneous injection. The dog tissues were subjected to gross and microscopic examinations and immunohistochemical analysis and the dogs’ serum cardiac troponin-I (cTn-I) was measured.

**Results:**

Systemic hemorrhage and/or congestion were the most marked pathological changes in both the naturally and experimentally PRV-infected dogs. Macroscopically, the major lesions consisted of petechiae and ecchymoses in both the endocardium and epicardium, thrombi in the mitral valves, hemorrhage in the lungs and thymus, and incomplete contraction of the spleen. Microscopically, the major histopathological findings were systemic hemorrhage and congestion, nonsuppurative ganglioneuritis (in the experimentally infected dogs, unexamined in the naturally PRV-infected dogs), brainstem encephalitis (in the naturally infected dogs), necrosis or exudation in the myocardium, and lymphoid depletion in many lymphoid organs and tissues. Viral antigens were only detected in the brainstems and peripheral ganglia of the infected dogs. Serum cTn-I was significantly higher in the experimentally PRV-infected dogs with myocardial lesions than in the dogs without myocardial lesions.

**Conclusions:**

Based on these results, we conclude that virally induced systemic hemorrhage, peripheral nervous system pathology, and/or cardiac injury can individually or collectively cause death in PRV-infected dogs. The respiratory signs of the disease are attributed to cardiogenic lesions.

## Background

Pseudorabies (also called “Aujeszky’s disease”) is an acute, frequently fatal disease caused by *Pseudorabies virus* (PRV), which belongs to the genus *Varicellovirus*, in the *Alphaherpesvirinae* subfamily of the *Herpesviridae* [1]. Pigs are the main reservoir of PRV, but many mammals are also susceptible to this infection [2,3]. It is believed that dogs (both farm dogs and companion dogs), can be infected with this virus by consuming contaminated raw pork or offal [4-6].

The clinical manifestations of canine pseudorabies differ from those of swine pseudorabies. Localized pruritus occurs in canines, but is often absent in older swine [7]. The incubation time is 2–9 days in dogs. Most infected dogs die within 48 h of the onset of symptoms [8]. The clinical symptoms are similar among dogs, including facial pruritus, dyspnea, vomiting, bloody diarrhea, edema, ataxia and muscle spasms [7,9,10]. However, it is not unusual for some dogs to die without showing any of the typical symptoms [11].

Very few reports of the histological lesions in PRV-infected dogs are available, and histopathological studies have been limited to the heart and nervous system [4,9,12]. However, the systematic pathological characteristics of canine PRV infection are not well understood.

Cardiac troponin-I (cTn-I) is a cardiac biomarker detectable in the circulation after cardiomyocyte death or injury, regardless of the underlying cause [13]. cTn-I is released from injured myocardiocytes into the circulation within hours of injury, peaks within 2 d, and remains elevated for as long as the injury continues. Increased serum cTn-I can be used effectively to detect, monitor and quantify ongoing cardiac injury [14].

Since early 2011, the incidence of PRV infection has increased on pig farms in north China, among pigs that were previously vaccinated against PRV. This outbreak has affected more than nine provinces and municipalities, including Beijing [15,16]. The incidence of canine PRV infections increased simultaneously with this outbreak of swine pseudorabies [17,18]. In total, 13 cases of canine pseudorabies were identified in our laboratory during the period from December 2011 to October 2013, including in farm dogs and pet dogs from rural areas and urban areas of Beijing. The pathological changes in these 13 naturally PRV-infected dogs were summarized in this study to provide an update on the systematic pathological characteristics caused by the PRV distributed in north China. We also conducted an experimental study in which dogs were infected experimentally with PRV isolate BJ-YT to investigate the pathogenesis of PRV infection in dogs.

## Results

### Naturally PRV-infected dogs

PRV infection was confirmed with PCR, immunohistochemistry, virus isolation, and a rabbit inoculation test (see Table 1).

**Table 1:** **Diagnostic laboratory test results for naturally PVR-infected dogs**

**Dog number**	**1**	**2**	**3**	**4**	**5**	**6**	**7**	**8**	**9**	**10**	**11**	**12**	**13**
PCR	+	+	+	+	+	−	−	+	+	+	+	−	−
Immunohistochemistry	NA	NA	+	+	+	+	+	+	+	+	NA	NA	NA
Rabbit inoculation test	NA	NA	NA	NA	+	+	+	NA	NA	NA	NA	NA	NA
Virus isolation	NA	NA	NA	NA	+	+	−	+	+	+	+	+	+

+, positive; −, negative; NA, not available.

The dogs showed pruritus (13/13, 100%), progressively worsening tachypnea and dyspnea (11/13, 85%), hypersalivation (10/13, 77%), hematemesis (4/13, 31%), tremor (4/13, 31%) and emesis (2/13, 15%). No obvious abnormalities were observed on thoracic radiographs (4/4, 100%). 9 of the 13 (69%) dogs had a history of consuming raw pork or offal, according to their owners.

**Figure 1:**
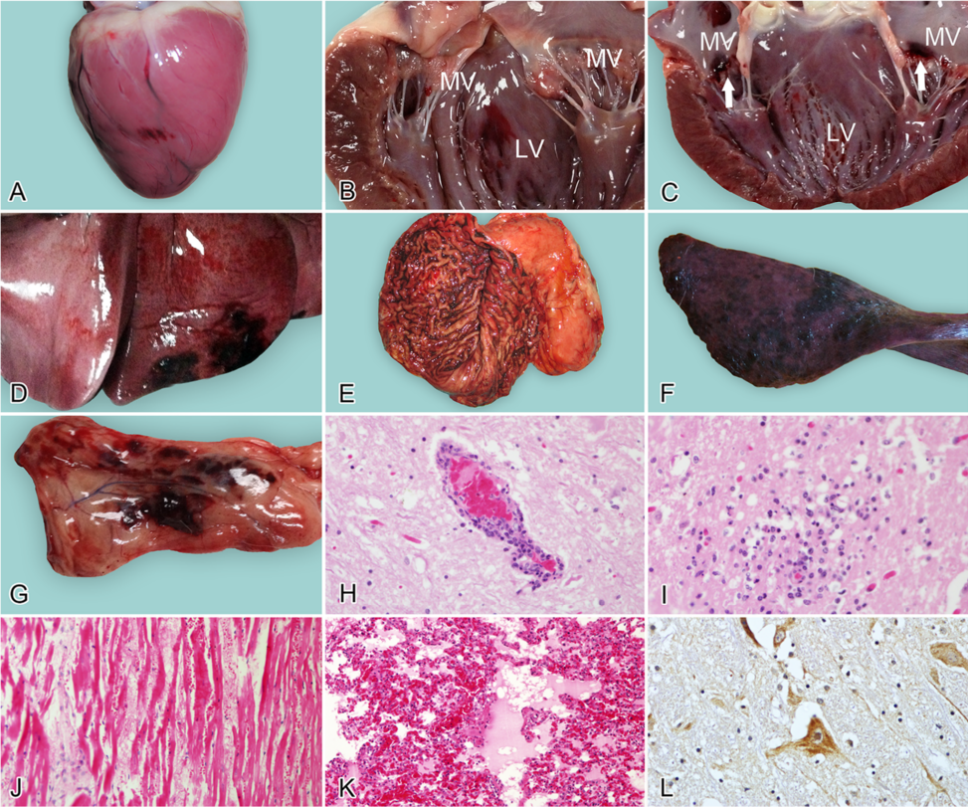
**Gross and microscopic lesions in naturally PRV-infected dogs. A**: Heart, showing ecchymoses in the epicardium; **B**: heart, showing an ecchymosis in the papillary muscle of the left ventricle (LV, left ventricle; MV, mitral valves); **C**: heart, showing focal hemorrhage (arrows) in the mitral valves (LV, left ventricle; MV, mitral valves); **D**: lung, focal hemorrhage; **E**: stomach, diffuse deep-red discoloration of the gastric mucosa as a consequence of congestion and hemorrhage; **F**: spleen, numerous dark-red to black, raised, soft, blood-filled areas of various sizes are incompletely contracted areas; **G**: thymus, evident hemorrhage; **H**: brainstem, showing perivascular cuffing around a small blood vessel (HE); **I**: hyperplasia of glial cells (HE); **J**: cardiac muscle, vertical section, in which the parallel arrays of myofibers are disrupted by fibrin and erythrocytes; note the myocardial fiber degeneration, necrosis with hypereosinophilia, and loss of cross-striations (HE); **K**: lung, hemorrhage and exudation in the lung (HE); **L**: brainstem, immunohistochemical detection of PRV antigen in the brainstem. DAB was used as the chromogen.

Gross abnormalities were recorded at necropsy. Cardiac abnormalities (11/13, 85%) typically included epicardial hemorrhage (5/13, 38%; Figure 1A), endocardial hemorrhage (9/13, 69%; Figure 1B), valvular hemorrhage (3/13, 23%; Figure 1C), and cardiac thrombi (3/13, 23%). Focal pulmonary hemorrhage and/or congestion (11/13, 85%; Figure 1D) were the most common findings in the respiratory system, and one dog had frothy fluid in the airways. Gastric hemorrhage (6/9, 67%; Figure 1E) was also observed. The splenic pathology was particularly uniform, characterized by numerous dark-red to black, raised, soft, blood-filled areas of various sizes (9/13, 100%; Figure 1F). Thymic hemorrhage (11/11, 100%; two dogs were excluded because their advanced age meant that their thymuses were unavailable; Figure 1G) and renal hemorrhage (2/13, 15%) were also observed. Other gross lesions included subcutaneous edema (2/13, 15%) and pleural hemorrhage (1/13, 8%).

Because the literature on canine PRV and our previous experience of it are limited, only some tissues from the 13 dogs were examined microscopically. The lesions in the brainstem were the most significant. Nonsuppurative encephalitis (8/8, 100%; Figure 1H, I) consisted of mild-to-severe perivascular cuffing, and glial proliferation and neuronophagia were observed in the brainstem. Lesions in the cerebrum and cerebellum were limited, and only mild congestion (1/5, 20%) was noted. Mild-to-severe hemorrhage with noninflammatory, eosinophilic fiber-like exudate in the stroma (3/6, 50%; Figure 1J) was the most obvious cardiac change. Moderate-to-severe pulmonary edema, congestion, and/or hemorrhage (3/3, 100%; Figure 1K) were also observed. The pathological changes in the liver included mild-to-severe congestion (6/6, 100%) and focal necrosis (3/6, 50%). The thymus showed diffuse moderate-to-severe hemorrhage (6/6, 100%) and lymphoid depletion (3/6, 50%). Incompletely contracted areas of the spleen (3/4, 75%) were also observed. The lymph nodes showed lymphoid depletion (4/4, 100%) and hemorrhage (2/4, 50%). Acute nephritis was typical, consisting of moderate-to-severe interstitial congestion (5/5, 100%) and the accumulation of protein fluid in the renal tubules and capsule (3/5, 80%).

PRV antigen was detected in the brainstem (Figure 1L).

### Experimentally PRV-infected dogs

The incubation period ranged from 87 to 93 h in the five dogs experimentally inoculated with PRV. All the infected dogs died within 31 h of the onset of symptoms. All dogs displayed depression, anorexia, pruritus, and vocalization. An intense, localized pruritus of the injected region lasted until death, and self-induced trauma to the skin was prominent in all the infected dogs. Aconuresis (3/5), progressively worsening tachypnea and dyspnea in the later stage (2/5), and hypersalivation (1/5) were noted. The control dogs showed no abnormalities during the study period.

### Gross pathology

In the experimental group, dog No. 5 was euthanized by intravenous anesthetic at the moribund stage on the fourth day post infection (DPI), whereas the other four dogs were euthanized at the moribund stage on DPI 5. The dogs in the control group were killed after the last dog in the PRV-infected group was killed.

**Figure 2:**
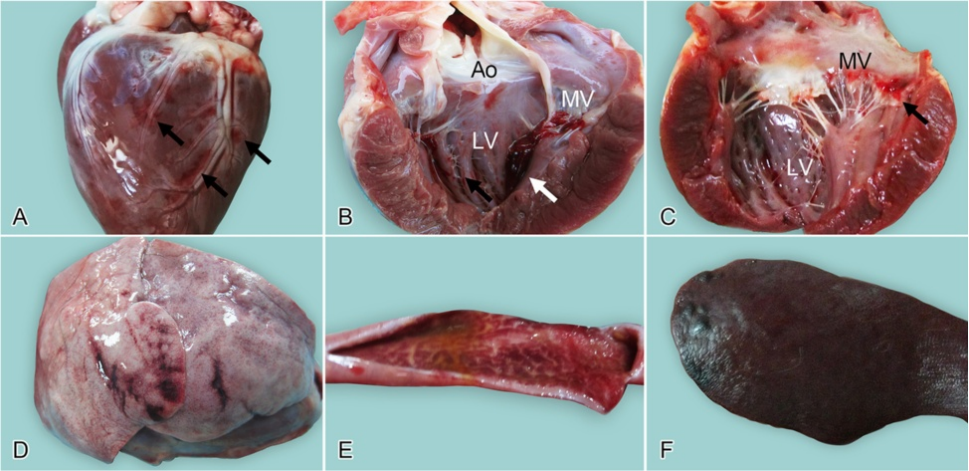
**Gross lesions in experimentally PRV-infected beagle dogs. A**: Heart, showing multifocal petechial hemorrhage in the epicardium; **B**: heart, a dark-red thrombus attached to the mitral valves (white arrow; note the ecchymosis [black arrow] present in the endocardium of the left ventricle (Ao, aorta; LV, left ventricle; MV, mitral valves); **C**: heart, showing extensive hemorrhages in the mitral valves (arrow) (LV, left ventricle; MV, mitral valves); **D**: lung, showing focal redness arising from congestion and hemorrhage; **E**: duodenum, diffuse redness of the mucosa resulting from hyperemia and congestion; **F**: spleen, incompletely contracted areas characterized by numerous dark-red to black, soft, blood-filled projections at the margins.

Macroscopic cardiac lesions were observed in three of the five PRV-infected dogs (Nos 1, 2, and 3). Two of these dogs (Nos 1 and 2) showed various degrees of petechiae and ecchymoses in both the epicardium (Figure 2A) and endocardium (Figure 2B), and two dogs (Nos 1 and 3) showed hemorrhage in the bicuspid valve (Figure 2C). Thrombi were seen in the mitral valves of one dog (No. 2; Figure 2B). Focal ecchymoses in the lung were observed in two dogs (Nos 1 and 2; Figure 2D). Diffuse deep-red discoloration of the gastric mucosa was observed in one infected dog. Focal or diffuse redness of the mucosa was present in the duodenum (3/5 Figure 2E) and jejunum (3/5). Irregular dark-red raised areas were noted in the spleen (4/5; Figure 2F). Subcutaneous edema over the underjaw (1/5) and clear ascites in the peritoneal cavity (1/5) were observed in the experimental group.

No gross lesions were observed in the other organs or tissue samples from the experimental group. No gross lesions were observed in the dogs in the control group.

### Histopathological findings

**Figure 3:**
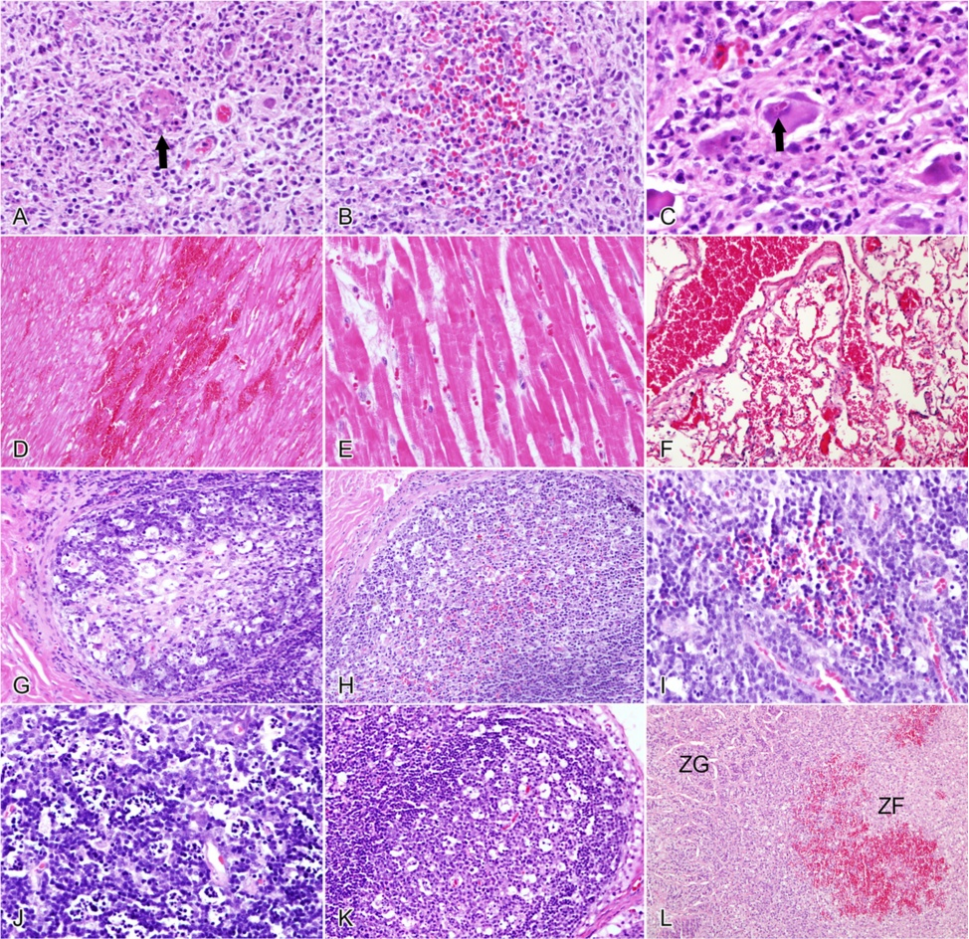
**Histopathological findings in experimentally PRV-infected beagle dogs. A**: Stellate ganglion, salient gliosis and neuronophagia (arrow), and nuclear debris from necrotic cells are frequently observed (HE); **B**: stellate ganglion, showing mild hemorrhage (HE); **C**: stellate ganglion, acidophilic intranuclear inclusion bodies (arrow) (HE); **D**: cardiac muscle, extensive myocardial hemorrhage and necrosis in the myocardium, swelling and vacuolization of the muscle fibers, loss of striation, and granular fibers are visible (HE); **E**: cardiac muscle, less-severe changes with the accumulation of eosinophilic fiber-like exudates in the myocardial interstitium (HE); **F:** lung, pulmonary hemorrhage and congestion (HE); **G**: ileum, lymphoid depletion in the lymphatic nodules (starry sky aspect) (HE); **H**: cecum, mild hemorrhage in the lymphoid nodules (HE); **I**: thymus, mild hemorrhage (HE); **J**: thymus, showing lymphoid-depleted areas (HE); **K**: mesenteric lymph nodes, showing lymphoid depletion (HE); **L**: adrenal gland, hemorrhage in the zona fasciculata, ZG, zona glomerulosa; ZF, zona fasciculata (HE).

In the PRV-infected group, moderate-to-severe, nonsuppurative ganglioneuritis, characterized by multifocal areas of necrosis, pronounced gliosis, and neuronophagia (5/5; Figure 3A), hemorrhage (4/5; Figure 3B), and eosinophilic intranuclear inclusion bodies (2/5; Figure 3C) were observed in the stellate ganglion. Nonsuppurative ganglioneuritis was also noted in the celiac ganglion (1/5).

Three of the PRV-infected dogs (Nos 1, 2, and 5) had microscopic lesions in their myocardium. Extensive foci of myocardial hemorrhage and necrosis (Figure 3D) were observed in two dogs (2/5, Nos 1 and 2). The sarcoplasm of the affected myocardiocytes was swollen, granular, and deeply eosinophilic. Necrotic myocardiocytes lost their cross-striation and became fragmented. There was no or minimal inflammatory reaction. One dog (No. 5) accumulated an eosinophilic fiber-like exudate in the myocardial interstitium (Figure 3E), although no hemorrhage was observed. Four PRV-infected dogs showed mild-to-moderate pulmonary congestion. Leakage of erythrocytes and plasma proteins was observed in the lung of 1 dog (No.2; Figure 3F).

Mild-to-moderate congestion in the small intestine, mild-to-salient lymphoid depletion in the lymphatic nodules of the ileum (3/5; Figure 3G), cecum (2/5), and colon (4/5), and mild hemorrhage in the lymphoid nodules of the ileum (3/5), and cecum (2/5; Figure 3H) were the most frequent observations in the alimentary tracts of the PRV-infected dogs. Necrosis of the gastric mucosa was observed in one PRV-infected dog.

The spleens of the PRV-infected dogs displayed moderate-to-severe incomplete contraction. The incompletely contracted parenchyma was filled with blood, although the intervening tissues were normal, and the contracted splenic red pulp was devoid of blood. The thymus showed mild-to-moderate hemorrhage (4/5; Figure 3I) and significant lymphoid depletion (4/5; Figure 3J) compared with the controls. The mandibular lymph nodes displayed mild hemorrhage (4/5) and the mesenteric lymph nodes showed lymphoid depletion (5/5; Figure 3K).

Mild-to-moderate hemorrhage in the inner cortex (zonae fasciculata and reticularis) of the adrenal glands was noted in all the PRV-infected dogs (5/5; Figure 3L).

No histopathological aberrations were detected in the other organs or tissue samples. No microscopic lesions were observed in the control group.

### Immunochemistry

**Figure 4:**
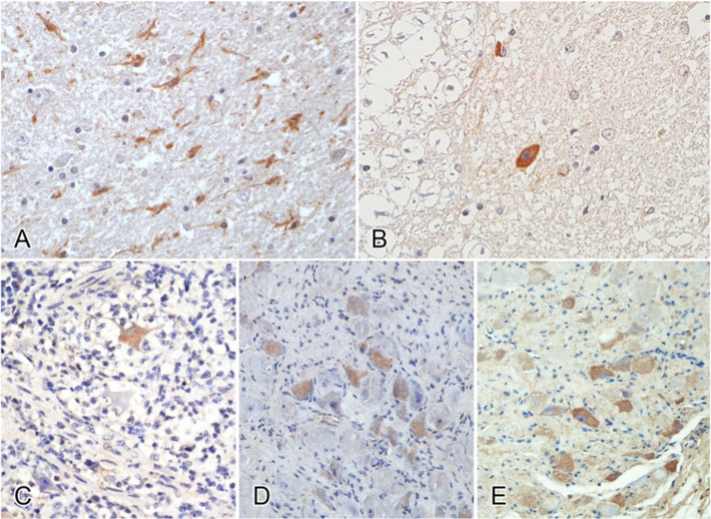
**Immunohistochemical detection of PRV antigen in experimentally PRV-infected beagle dogs. A**: Brainstem; **B**: cervical spinal cord; **C**: stellate ganglion; **D**: celiac ganglion; **E**: caudal mesenteric ganglion. DAB was used as the chromogen.

Productive infection of the neurons in the nervous system was confirmed with immunochemical staining. PRV antigen was detected in the brainstem (4/5; Figure 4A), cervical spinal cord (2/5; Figure 4B), stellate ganglion (5/5; Figure 4C), celiac ganglion (5/5; Figure 4D), and caudal mesenteric ganglion (4/5; Figure 4E). No viral antigen was detected in the other tissues or organs.

### cTn-I analysis

**Figure 5:**
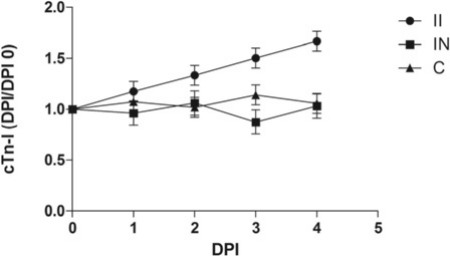
**Serum cTn-I concentration (least-squares mean ± SE) of the experimental beagle dogs.** II, Infected group with myocardial injury; IN, infected group without myocardial injury; C, control group.

To investigate the potential relationship between the cardiac lesions and respiratory signs, or even the deaths of the PRV-infected dogs, the serum concentrations of cTn-I in the experimental animals were evaluated. The three infected dogs with myocardial injury (group II; Nos 1, 2, and 5) showed increased cTn-I,whereas the infected group without myocardial injury (group IN; Nos 3 and 4) and control group (group C) showed only slight fluctuations (Figure 5).

Assessment of the interactions between the groups and DPIs revealed a significant overall difference between the II and IN groups (P < 0.05) and between the II and C groups (P < 0.05). The results of individual least-squares mean (LSM) comparisons revealed significant differences on DPI 2, DPI 3, and DPI 4 (on DPI 2: II vs C,P < 0.05; DPI 3: II vs C, P < 0.05; II vs IN, P < 0.05; DPI 4: II vs C, P < 0.001; II vs IN, P < 0.001) (see Table 2).

**Table 2:** **Serum cTn-I (LSM ± SE) in the experimental animals**

**DPI**	**II**	**IN**	**C**
1	1.18 ± 0.10	0.96 ± 0.12	1.07 ± 0.10
2	1.33 ± 0.10^a^	1.06 ± 0.12	1.02 ± 0.10
3	1.50 ± 0.10 ^a,b^	0.87 ± 0.12	1.14 ± 0.10
4	1.67 ± 0.10^A,B^	1.03 ± 0.12	1.06 ± 0.10

II, Infected group with myocardial injury; IN, infected group without myocardial injury; C, control group; ^a^compared with C, P < 0.05; ^b^compared with IN, P < 0.05; ^A^compared with C, P < 0.001; ^B^compared with IN, P < 0.001.

## Discussion

Systemic hemorrhage was the most prominent pathological finding in both the naturally and experimentally PRV infected dogs, although the hemorrhage in many tissues and organs was milder in the experimentally infected dogs than in the naturally infected dogs. This finding indicates that hypovolemic shock caused by systemic hemorrhage plays an important role in the pathogenesis of canine pseudorabies and causes the death of PRV-infected dogs.

Gross lesions of the nervous system were rarely observed in either the naturally or experimentally PRV-infected dogs. The histological findings in the central nervous systems (CNSs) of the naturally PRV-infected dogs were restricted to the brainstem, which is consistent with previous reports of PRV in dogs [4,9] and other unnatural hosts such as domestic cats [19] and foxes [20]. However, there were no CNS lesions in the experimentally infected dogs. In contrast, salient nonsuppurative ganglioneuritis was observed and PRV antigen was also detected in all the experimentally infected dogs. Similar findings have been reported in the ganglia of the peripheral nervous systems (PNSs) of mice [21,22]. Unfortunately, the peripheral ganglia of the naturally PRV-infected dogs were not examined in the present study. Some researchers have suggested that the observed hypersalivation may emanate from trigeminal ganglioneuritis [7,9,23,24], and that pruritus is caused by the infection of the trigeminal ganglia and dorsal root ganglia [21,22]. Wild-type PRV-infected animals also display a dramatic peripheral neuropathy and do not die of brain infection [21]. Based on these previous studies, it is possible that PRV induces a PNS pathology and impairs the sympathetic functions, leading to organ dysfunction and even death in the infected dogs.

Cardiac lesions were another prominent finding in both the naturally and experimentally PRV-infected dogs, consistent with other reports [4,7,12]. It is noteworthy that progressive tachypnea was frequently observed in the clinically PRV-infected dogs, which often showed cardiac lesions on autopsy. Therefore, we believe that the cause of tachypnea may be attributable to heart damage. To examine this notion, we measured the dogs’ serum cTn-I levels, because the cTn-I is highly specific for myocardial injury [25]. Three of the five experimentally PRV-infected dogs with myocardial injury showed elevated cTn-I levels when they were still alive. Statistical analysis of the cTn-I levels showed that cTn-I was significantly higher (P < 0.05) in the PRV-infected dogs than in the dogs without myocardial injury. These findings suggest that myocardial injury is a very important and specific lesion in PRV-infected dogs and that cTn-I can be a good reference indicator for the prognoses of dogs suspected of PRV infection. Valvular thrombi were observed in both the naturally and experimentally PRV-infected dogs. It is highly likely that the thrombi observed in this study were secondary to valvular injury and systemic hemorrhage, because cardiac thrombi are usually initiated by endothelial damage. Turbulence in the valvular areas of the heart simulates interactions between coagulation factors and therefore microthrombic adhesion [26]. It is highly possible that excessive sympathetic stimulation to the myocardium caused by the ganglioneuritis of the stellate ganglia and endothelial disruption, may initiate arrhythmia and even lead to sudden death [12]. It is noteworthy that lung macrophages have been identified as PRV target cells in swine [27], and that viral replication induces an enormous influx of phagocytes. Necrosis is also prominent in the lung tissues of infected swine [28], and the resulting massive tissue destruction causes respiratory signs, such as sneezing, coughing, nasal discharge, and dyspnea [29]. In the present study, rapidly worsening tachypnea and dyspnea in the later stage of infection were frequently observed in the naturally PRV-infected dogs and in two of the experimentally infected dogs with prominent cardiac lesions. However, neither viral antigen nor necrosis was detected in the lung tissues. Other studies of infected mice detected no PRV in the phrenic motoneurons of the spinal cord or in the respiratory center of the medulla oblongata [22,30]. Therefore, the acute death of unnatural hosts caused by PRV may not be attributable to neurally based respiratory failure. We believe that excessive sympathetic cardiac stimulation, associated with neuritis, ganglioneuritis, and cardiac injury, may lead to the accumulation of blood in the cardiac chamber and pulmonary edema/congestion, which are possibly caused by heart failure, thus producing cardiac asthma. Hence, it is highly likely that the respiratory signs in dogs are caused by neither primary pulmonary damage nor respiratory center infection, but by cardiogenic lesions. This may also explain why the PRV-infected dogs with progressive asthma/dyspnea showed no obvious pulmonary abnormalities on clinical thoracic radiographs.

Incomplete contraction of the spleen was observed in all the naturally and experimentally infected dogs. This results from the failure of the smooth muscle to contract in some areas, and is caused by sympathetic disfunction or circulatory shock, as occurs in “fight or flight” situations [26].

Lymphoid cells were reduced in the thymus and lymph nodes of both the naturally and experimentally infected dogs. Hyperplasia of the lymphoid organs has been observed in PRV-infected foxes [20], although it was not observed in the present study. The lack of hyperplasia in the lymphatic system may cause widespread damage. Early researchers in PRV infection speculated that the infected lymphocytes of swine provide an alternative route for the transmission of the virus [31]. However, we detected no PRV antigen in the lymphoid organs or tissues of these dogs.

Hepatonecrosis was also observed in the naturally PRV-infected dogs, but not in the experimentally PRV-infected dogs. This could be attributable to the fact that in canines, foreign particles are predominately trapped and removed by Kupffer cells in the liver, which would result in vastly excessive cytokine production [32]. Therefore, the naturally PRV-infected dogs were more susceptible to liver injury, possibly because they had been exposed to various pathogens during their lives. The naturally PRV-infected dogs also tended to display acute nephritis, whereas the experimentally infected dogs did not. The reason for this discrepancy is unclear. It appears that nephritis is attributable to the deposition of immune complexes, because immune complex nephritis is often associated with the loss of filtration selectivity, so that the protein-containing contents of the tubules cause their lumens to dilate [33]. Another possible explanation is that PRV cross-reacts with an autoantigen.

In this study, adrenal hemorrhage was confined to the cortex, particularly to the zona reticularis, the area stimulated by adrenocorticotropic hormone. This phenomenon has also been reported in the mink [34]. Immunohistochemical staining for viral antigen also revealed that many sympathetic postganglionic neurons (stellate, celiac, and caudal mesenteric ganglion) were infected. However, no viral antigen was detected in the adrenal gland, even though the adrenal medullary parenchymal cells are modified sympathetic neurons. Why the adrenal cortex is sensitized to hemorrhage is not easily explained, and the significance of this lesion is unclear.

A few early reports indicated that PRV often fails to produce gross lesions in dogs [35-37], whereas other studies have suggested that pathological changes seem to become more serious with time [29,38]. This phenomenon may be associated with viral virulence and/or the physical status of the infected dog. For example, significantly more systemic hemorrhage was observed in our preliminary study (unpublished) than in the present study, and these differences were associated with the different batches of beagle dogs used for the experiments.

In swine, PRV is transferred to various organs by viremic and lymphatic pathways [39]. However, this is not the case in dogs, because no evidence of viral replication was found in the tissues of the experimentally infected dogs, except in the nervous system. Therefore, we infer that the non-neural tissue damage in the infected dogs is induced indirectly by PRV.

Dogs are thought to be infected by PRV either through the consumption of raw meat or offal from swine, or by contact with infected swine or swine carcasses [6,7,9]. However, in the present study, four of the 13 naturally PRV-infected dogs had no history of direct contact with swine or swine carcasses. Although the air-borne transmission of PRV, even over long distances, is possible between swine [40,41], it has not been reported in dogs. An early report suggested that sheep, another unnatural host, are probably infected through skin abrasions [42]. Therefore, it is possible that contact with contaminated garbage or food through an injured alimentary tract is a possible route of infection in dogs. Injuries acquired while chewing something hard may increase the susceptibility of dogs to infection when they are exposed to a PRV-contaminated environment.

In China, PRV has not yet been eradicated from domestic swine herds. Therefore, it may be transmitted freely among different vertebrate species, including dogs. Further studies of the pathogenesis of canine PRV infection could improve its diagnosis and allow the prevention of PRV infection in dogs and other domestic animals.

## Conclusions

Based on our study results, we conclude that virally induced systemic hemorrhage, PNS pathology, and/or cardiac injury individually or collectively cause the death of PRV-infected dogs. Cardiogenic lesions are responsible for the respiratory signs observed in PRV-infected dogs.

## Methods

There were two data sources were used in this study: naturally PRV-infected dogs and experimentally PRV-infected dogs.

### Study of naturally PRV-infected dogs

Thirteen naturally PRV-infected dogs were diagnosed and maintained in our laboratory at the Veterinary Teaching Hospital, China Agricultural University, Beijing, during the period from December 2011 to October 2013. Pseudorabies was first suspected based on their clinical histories, symptoms, and gross and histological lesions. Brainstem samples were collected from these dogs at necropsy, and a conventional PCR targeting a fragment of the PRV gB gene was used to diagnose the disease. The brainstems were also used for virus isolation on Vero cells (African green monkey kidney cells), immunohistochemical staining with an anti-PRV monoclonal antibody, and the rabbit inoculation test [43] to confirm the presence of PRV in the dogs. In the rabbit inoculation test, rabbits were inoculated subcutaneously with 1 mL of brainstem suspension from the suspected PRV-infected dogs. PRV infection was confirmed if the rabbits showed pruritus. The gross and histopathological changes were evaluated in each dog.

### Study of experimentally PRV-infected dogs

#### Ethics statement

The animal studies were approved by the Beijing Association for Science and Technology (approval reference SYXK [Beijing] 2007-0023). The experimental study was conducted in accordance with a study protocol (CAU-AEC-2010-0603) approved by the Animal Ethics Committee of China Agricultural University.

### Virus and experimental animals

The virus used in the experimental study was isolated from a Yorkshire terrier, one of the 13 naturally PRV-infected dogs, which died after it was fed with raw swine bones purchased from a local market. The virus was isolated on Vero cells and designated BJ-YT (GenBank: KC981239). Sequence analysis of the gE gene indicated that the virus shared 100% nucleotide identity with the swine PRV strain HB/HD, Hebei/05/2012 (GenBank: KC415027).

Eight healthy, 1-year-old, vaccinated (against canine parvovirus, canine distemper, rabies, canine adenovirus, canine parainfluenza, and leptospira) beagle dogs (four males, four females) were purchased from Beijing Keyu Experimental Animal Breeding Center, and were confirmed to be serologically negative for PRV antibodies with a microtitration serum neutralization test. The animals were randomly allocated to two groups on arrival at the laboratory. Five of the dogs (Nos 1, 2, 3, 4, and 5) were allocated to the experimentally PRV-infected group and received 2 × 10^6^ TCID_50_ of PRV isolate BJ-YT in 2 mL by subcutaneous inoculation under anesthesia induced with an intramuscular injection of Zoletil 100 (tiletamine–zolazepam, Virbac, Carros, France). The remaining three dogs (Nos 6, 7, and 8) received 2 mL of Dulbecco’s Modified Eagle’s Medium by subcutaneous injection, and were used as the negative control group. The two groups were housed separately, and each dog was caged individually throughout the experimental period, and fed a commercially available dog food once a day.

The animals were inspected visually at least four times daily for clinical signs of disease. The body temperatures of the animals were recorded twice a day, in the morning and afternoon. The animals were euthanized in the agonal stage and subjected to detailed necropsy.

### cTn-I analysis

Blood samples were collected from the veins of the dogs by direct venipuncture into sterile tubes on Day 0 (the day before the experimental PRV infection was initiated) and on each DPI until necropsy (DPIs 1–5). The blood samples were allowed to clot and were centrifuged (1000 × g for 15 min at 4–8°C), the supernatants were collected, aliquoted, and stored deeply frozen (approximately at -80°C) in cryotubes until analysis. The serum samples were thawed immediately before the cTn-I analysis.

To investigate the correlation between the cardiac lesions and the respiratory signs or even the deaths of the PRV-infected dogs, the serum concentrations of cTn-I were measured with a commercial sandwich enzyme-linked immunosorbent assay kit (Canine Cardiac Troponin-I (cTn-I) ELISA Kit; ShangHai MEIXUAN Biological Science and Technology Ltd, Shanghai, China), according to the manufacturer’s instructions. For each dog, the ratio of the cTn-I concentration on each DPI was calculated relative to the cTn-I concentration on Day 0 and compared day by day and between animals.

Statistical analyses were performed with SAS v9.2 (SAS Institute). Two-way repeated analysis of variance was used to assess the changes in cTn-I, using the group (infected group with myocardial injury, II; infected group without myocardial injury, IN; control group, C) and DPI as the independent factors, and time as the repeated measure. The significance of the differences among the groups at each DPI (from DPI 1 to DPI 4) was tested. A value of P < 0.05 was considered statistically significant.

### Tissue sampling

All the animals were subjected to detailed necropsy immediately after euthanasia. A full macroscopic examination of their tissues was performed. The tissue samples required for histological examination were collected from the cerebrum, cerebellum, brainstem, cervical spinal cord, thoracic spinal cord, lumbar spinal cord, stellate ganglion, celiac ganglion, caudal mesenteric ganglion, vagus nerve, cardiac muscle, lung, liver, stomach, duodenum, jejunum, ileum, cecum, colon, rib, thymus, spleen, tonsils, mandibular lymph nodes, mesenteric lymphoid nodes, kidney, and adrenal gland. The samples were fixed in 10% neutral-buffered formalin, and then dehydrated and embedded in paraffin using standard laboratory procedures.

### Light microscopic examination and immunohistochemistry

The paraffin-embedded tissue samples were sectioned to 4 μm and stained with hematoxylin-eosin (HE). For immunohistochemical studies, the sections were examined for the presence of PRV antigen with a horseradish peroxidase (HRP) method using a primary HRP-conjugated anti-PRV mouse monoclonal antibody (provided by Prof. Hanchun Yang, Key Laboratory of Animal Epidemiology and Zoonosis, China Agricultural University, China). The antigen–antibody complexes were examined with the Polink-2 Plus HRP Detection Kit (GB-BIO, Beijing, China), according to the manufacturer’s instructions. The sections were incubated with diaminobenzidine (DAB; ZSGB-BIO, Beijing, China) for visualization. Finally, the slides were counterstained with hematoxylin. Sections of brainstem from a naturally PRV-infected dog were used as the positive control for each series of stained sections. Sections of brainstem from a healthy dog were included as the negative control.
